# Phonon Transport in Defect-Laden Bilayer Janus PtSTe Studied Using Neural-Network Force Fields

**DOI:** 10.1021/acs.jpcc.4c02454

**Published:** 2024-06-22

**Authors:** Lijun Pan, Jesús Carrete, Zhao Wang, Georg K. H. Madsen

**Affiliations:** † Department of Physics, Guangxi University, Nanning 530004, China; ‡ Institute of Materials Chemistry, TU Wien, 1060 Vienna, Austria; § Instituto de Nanociencia y Materiales de Aragón (INMA), CSIC-Universidad de Zaragoza, E-50009 Zaragoza, Spain

## Abstract

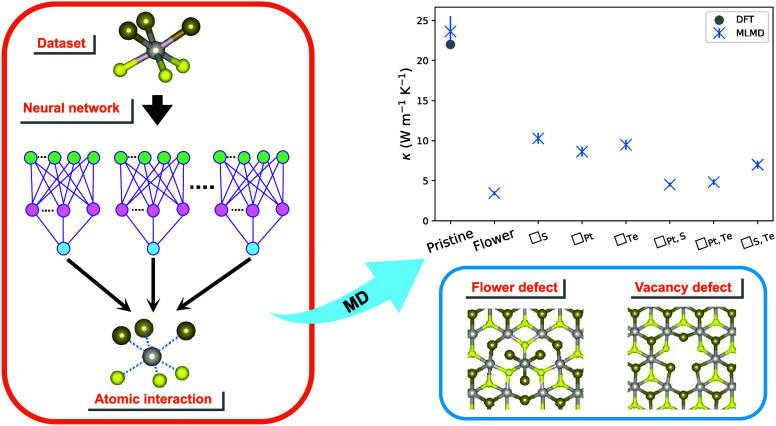

We explore the phonon transport properties of defect-laden bilayer PtSTe using equilibrium molecular dynamics simulations based on a neural-network force field. Defects prove very efficient at depressing the thermal conductivity of the structure, and flower defects have a particularly powerful effect, comparable to that of double vacancies. Furthermore, the conductivity of the structure with flower defects exhibits an unusual temperature dependence due to structural instability at high temperatures. We look into the distortion to normal modes around the defect by means of the projected phonon density of states and find diverse phenomena including localized modes and blue shifts.

## Introduction

Two-dimensional (2D) crystals often feature defects, which can be accidentally or deliberately introduced during their synthesis through various methods.^1^ Investigating the impact of those defects on phonon transport in 2D materials is crucial not only to comprehending the physics of thermal transport under realistic conditions^2,3^ but also to finding optimal candidate systems for applications such as thermoelectric and photoelectric devices, as well as thermal transistors.^1,4^

Within the extensive family of 2D materials, layered transition-metal dichalcogenides (TMDCs) have attracted considerable attention, particularly in the realm of thermoelectric applications, owing to their low thermal conductivity. This is exemplified by materials such as MoTe_2_^5^ and WTe_2_.^6^ Several reports reveal that point defects in TMDCs are intrinsic and significantly influence phonon transport properties, as observed in defect-laden monolayers of, e.g., MoS_2_ and WS_2_.^7−9^ For the purposes of phonon scattering, a point defect cannot be reduced to a single missing or replaced atom; it comprises the whole environment affected by that modification. For instance, in graphene, the impact of vacancies and other point defects on out-of-plane phonons, the dominant heat carriers, has been shown to be mediated mainly by changes in the local environment around those defects.^10^ However, studies have predominantly concentrated on monolayers. The behavior of defects in emerging multilayer TMDC systems may exhibit distinct characteristics and thus warrants further exploration.

The computation of thermal transport properties of defect-laden multilayered 2D crystals poses a substantial challenge for state-of-the-art ab initio methodologies. Structural defects disrupt the regular atomic arrangements, leading to a significant expansion in the size of the simulation box, especially when considering realistic defect concentrations. That is very detrimental for ab initio methods, which exhibit limited scalability in this respect.

**Figure 1:**
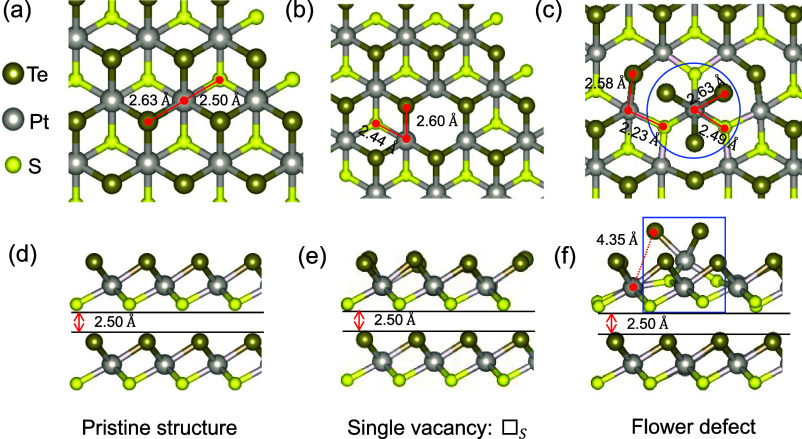
Optimized structures of pristine and defect-laden bilayer PtSTe: top view (a–c) and side view (d–f). The bond and interlayer distances are highlighted in red, and the flower defect is delimited in blue.

Inspired by recent successful applications of machine-learning force fields (MLFFs) to study phonon transport in several regimes,^11−13^ to overcome this challenge while preserving precision, we employ an accelerated Green–Kubo method in conjunction with molecular dynamics (MD) simulations based on an MLFF.^14−16^ Those simulations enable the exploration of the thermal transport properties of multilayer Janus PtSTe, a TMDC featuring broken out-of-plane symmetry and low thermal conductivity.^17^ Our chosen approach has previously demonstrated notable accuracy and efficiency in determining the thermal conductivity of van der Waals heterostructures, as illustrated in our prior work.^18^ In this paper, we systematically investigate phonon transport properties of defect-laden bilayer PtSTe, focusing on defects affecting a single layer in bilayer systems, as illustrated in Figure 1, to avoid the impact of interdefect coupling on the thermal conductivity as a confounding factor.

## Methods

### Defect Structures

Diverse structural defects can be present in 2D materials, each causing distinct effects on thermal transport properties. As mentioned above, vacancies are definitely interesting in this regard. We, therefore, include single and double vacancies (denoted as □_*X*_, where *X* represents the vacant site) among the defects in bilayer PtSTe we study here. The relaxed structure of □_S_ is depicted in Figure 1b,e. The optimized lattice parameter of pristine bilayer PtSTe is 3.80 Å, consistent with (19). The interlayer distance is 2.50 Å. Bond lengths change slightly upon the introduction of defects; details are given in the figure.

Another intriguing class of structural imperfections comprises topological defects. The best known of those are the Stone–Wales defects in graphene that arise from the rotation of a single C–C bond by 90°.^20^ Rotating a larger hexagonal domain within the graphene sheet can form a flower-like structure, which can reduce the high thermal conductivity of graphene by 1 to 2 orders of magnitude.^21^

Similar rotational defects have been identified in layered TMDCs.^22^ The generation of such defects in TMDCs involves rotating a copy of the motif by 60°, resulting in a 2D flower-like lattice pattern in TMDCs. This rotation in Janus PtSTe preserves the heteroatomic nature of bonding and the rotational symmetry, as illustrated in Figure 1c,f. Understanding the role of flower defects in the thermal transport of layered TMDCs is more intricate compared to graphene, primarily due to the polar nature of chemical bonds. This aspect has received particular attention in our study, as it remains poorly understood in the current scientific literature.

### Computational Approach

In some large or complex 2D systems, such as multilayer GeS,^23^ high-order phonon scattering plays a significant role in phonon transport. Consequently, ab initio computations of their thermal transport properties become prohibitively expensive, given the need to calculate higher-order interatomic force constants (IFCs) to achieve accurate predictions. As an alternative, MD simulations can handle large systems with computational efficiency. On the other hand, the precision of this method is heavily reliant on the choice of the force field model.

In the past decade, MLFFs have been developed to overcome this obstacle by learning from first-principles data, bridging the gap between ab initio and MD calculations.^24−26^ MLFFs have emerged as an effective tool for studying thermal transport properties in defect-laden crystals and demonstrated high accuracy.^27−29^ We specifically use a neural-network force field (NNFF)^30,31^ to reconstruct the potential energy hypersurface of the crystal and subsequently predict its thermal conductivity.

**Figure 2:**
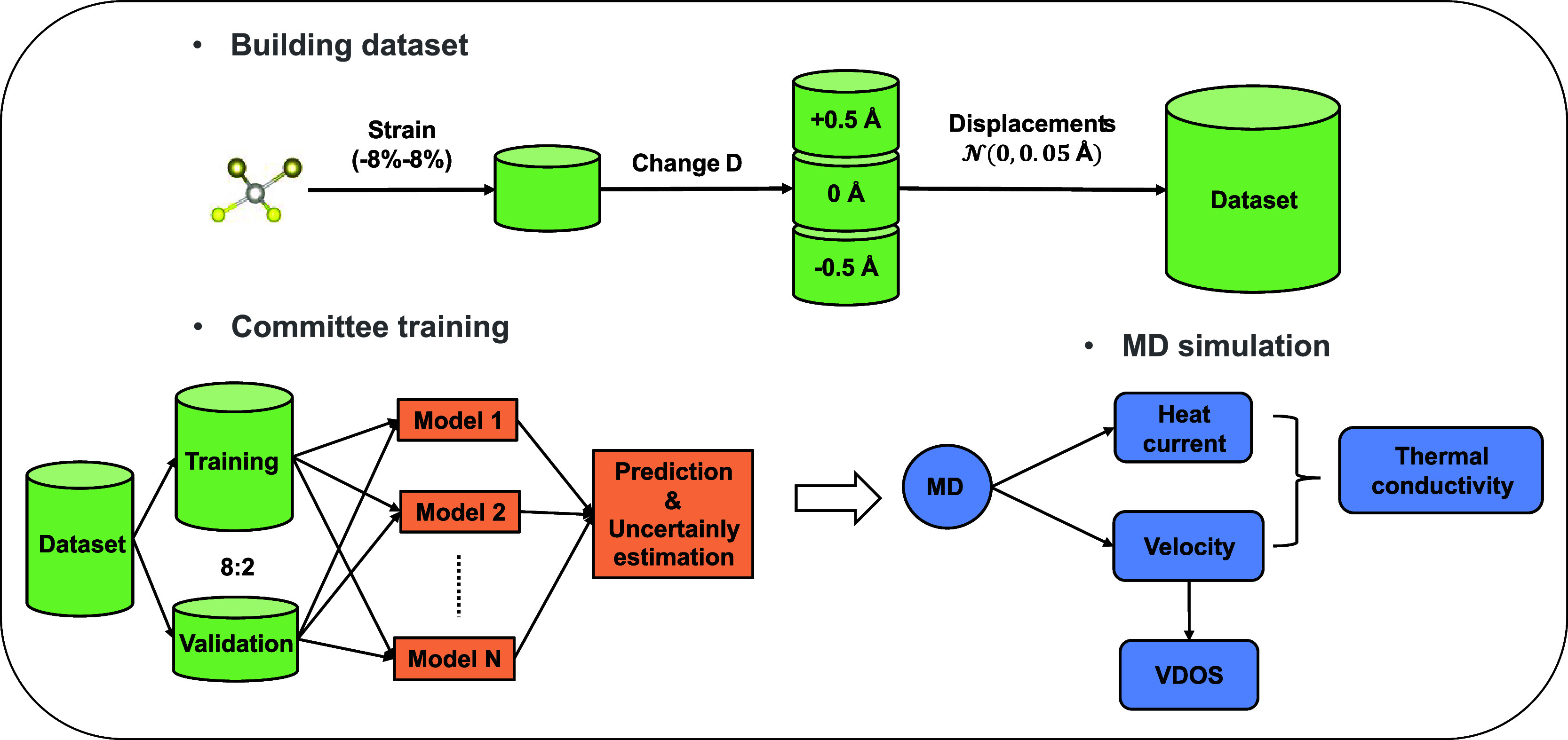
Schematic depiction of our workflow, including the training of the NNFF committee and the MD simulations.

We start the process by constructing an ab initio data set for training the NNFF, as illustrated in Figure 2. Subsequent to evaluating the accuracy of the trained NNFF, we apply it in MD simulations to determine the thermal conductivity of the specified defect-laden structures. The Green–Kubo method is chosen for this purpose. Our primary goal is to comprehend how various types of defects influence the computed thermal conductivity. Additionally, we conduct an analysis of the vibrational density of states (VDOS) to gain insights into the mechanisms governing the impact of defects on the vibrational spectrum. The theory and techniques employed at each step are detailed below.

### Data Set

The data set comprises atomic structures, potential energy, and atomic forces of pristine and defect-laden layered PtSTe, obtained from single-point ab initio calculations. Each defect-laden configuration contains a single instance of one of the classes of impurities included in this study: □_S_, □_Pt_, □_Te_ (single vacancies); □_Pt,S_, □_Pt,Te_, □_S,Te_ (double vacancies), or flower defects. To build the data sets, we start with pristine PtSTe. We first apply biaxial strain from −8 to 8% and then adjust the interlayer distance (*D*) within the range of −0.5 to +0.5 Å. Finally, a small random displacement drawn from a Gaussian distribution of *N* (0, 0.05 Å) is applied to the atomic positions. This process is illustrated in Figure 2.

Based on this, we generate 1200 distinct structures for the pristine system, varying the supercell size and including mono-, bi-, and trilayer configurations. Similarly, we also introduce 1000 defect-laden structures including single and double vacancies, as well as flower defects. In total, we obtain 2200 configurations, as detailed in Table 1.

**Table 1:** Composition of the Dataset

system	layers	supercell	number of configurations	run time per data point (min)
pristine	1	5 × 5 × 1	700	6
pristine	2	3 × 3 × 1	400	8
pristine	3	3 × 3 × 1	100	20
single vacancy	2	4 × 4 × 1	350	13
double vacancies	2	4 × 4 × 1	150	13
flowers	2	4 × 4 × 1	500	13

Ab initio calculations are carried out to compute the energy of and forces in these structures within the framework of Kohn–Sham density functional theory (DFT), as implemented in the Vienna ab initio simulation (VASP) package.^32^ The exchange-correlation energy is treated within the Perdew–Burke–Ernzerhof (PBE) generalized gradient approximation (GGA).^33^ Van der Waals interactions are approximated using the DFT-D3 correction.^34^ We utilize a 5 × 5 × 1 Monkhorst–Pack *k*-point mesh and set the plane-wave cutoff energy to 520 eV, with a convergence threshold set to 10^–8^eV for the energy. The optimized bilayer PtSTe, with a vacuum layer of 25 Å, serves as our baseline structure. All calculations were run on the same 28-core CPU; Table 1 shows the average run time for each data point in every category as a practical indicator of their complexity.

### Machine-Learning Model

The data set is randomly split into training and validation sets with an 0.8:0.2 ratio for training and testing of the NNFF model, respectively. To estimate uncertainty, the NNFF model is trained five times with different initial parameters, collectively forming the kind of ensemble known as a committee model,^31^ as illustrated in Figure 2.

For the model input, the Cartesian coordinates of the nuclei are transformed into element-dependent spherical Bessel descriptors.^30,35^ We set a cutoff radius of 5.0 Å and a maximum radial order of 6 for those descriptors. Additionally, to describe the chemical nature of the central atom of each environment, we add element-based embedding coefficients to the inputs to the NNFF.^30^

The descriptors are used as inputs to the NNFF model, and the ab initio energy and forces are used as the reference for training. Our particular model, described in detail in (31), adopts a deep residual network (ResNet) architecture^36^ implemented on top of the JAX framework.^37^ The ResNet architecture is designed to address, through residual mapping and extensive use of layer normalization,^38^ the problem of vanishing gradients encountered in deep multilayer perceptrons, adding stability and improving the performance of the training process in the presence of higher numbers of hidden layers. In this application, we employ core widths of 64:32:16:16. We choose Swish-1 as our activation function.^39^ We train the model for 500 epochs, utilizing the fully nonlinear VeLO optimizer^40^ to expedite convergence. During training, the following loss function is minimized:
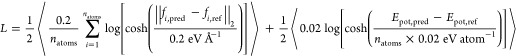
1where *f*_*i*_ and *E*_pot_ refer to potential energy and atomic forces, and the subscripts “pred” and “ref” indicate the values of those quantities in the predictions of the NNFF and according to the ground truth, respectively. The log-cosh function can be regarded as a smooth approximation to the absolute value function, and brings a degree of built-in gradient clipping to the training process when compared to the basic squared error often used to build the loss.

**Figure 3:**
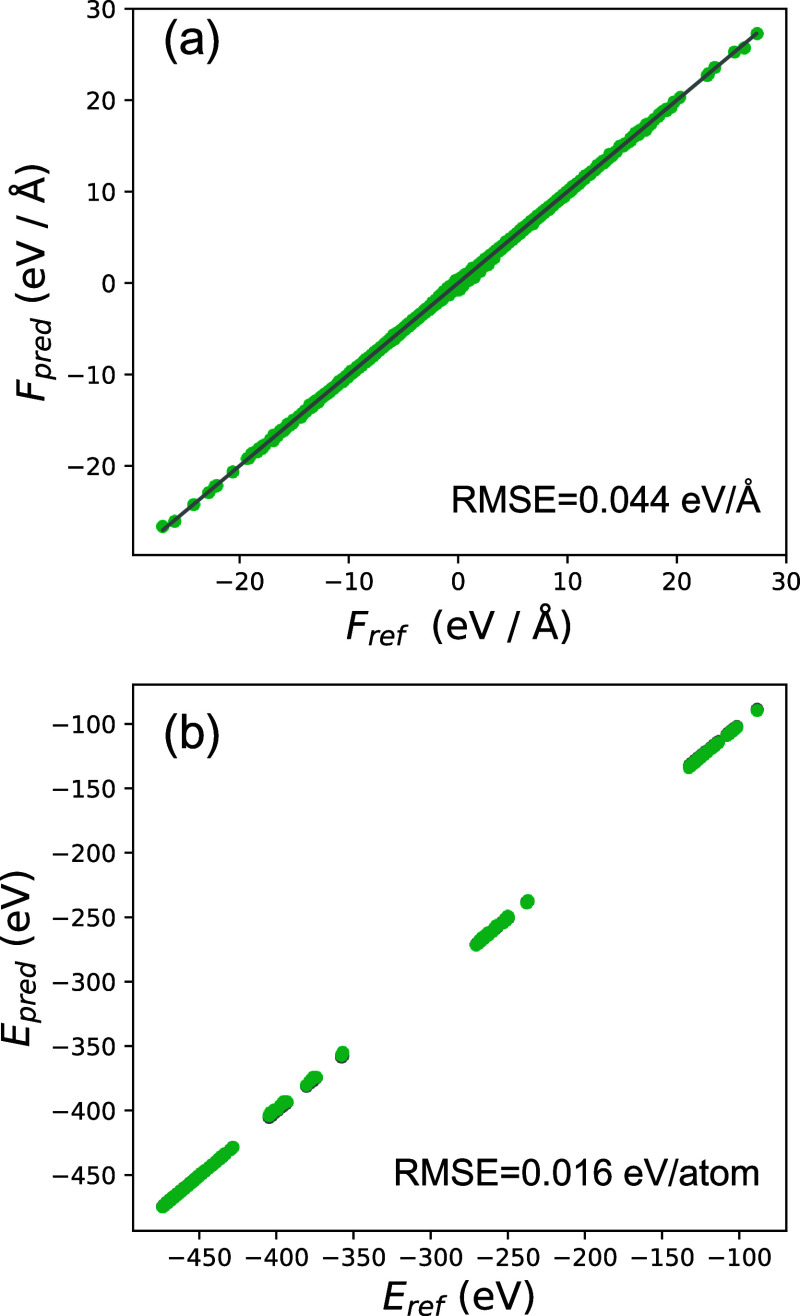
NNFF-predicted potential energy *E* (a) and atomic forces *F* (b) vs reference ab initio data.

In our NNFF, the potential energy is obtained as a sum of atomic contributions, and the forces are directly obtained from the automatic gradients of the energy function. The performance of our trained NNFF is evaluated against the ab initio energy and forces, as illustrated in Figure 3. It can be seen that the model exhibits a very high level of accuracy, achieving a root-mean-square error (RMSE) of 0.016 eV/atom and 0.044 eV/Å for per-atom energies and forces, respectively. After training, we generate an additional set of 360 defect-laden configurations to use as a separate test set. The RMSEs for energies and forces are found to be 0.021 eV/atom and 0.048 eV/Å, respectively, as shown in Figure S1 of the Supporting Information. The fact that those values are comparable to what we obtain for the validation set suggests that there is no significant overfitting. The MLFF dramatically speeds up the calculation of atomic forces and energies, for example, it only takes 0.31 s to process a data point for the pristine bilayer PtSTe, which is almost 4 orders of magnitude faster than the equivalent DFT calculation on similar hardware.

### MD Simulations

The NNFF is employed to conduct equilibrium MD simulations utilizing the atomic simulation environment (ASE).^41^ The in-plane lattice thermal conductivity of layered PtSTe structures featuring vacancies and flower defects is calculated following the Green–Kubo formalism.^42^ The process starts with an initial local energy minimization of each structure. After convergence tests, 250 ps simulations with a time step of 1.0 fs are carried out in the canonical (NVT) ensemble using Langevin dynamics to allow the system to attain thermal equilibrium at each given temperature. The simulations are then extended in the microcanonical (NVE) ensemble for >3000 ps to compute the atomic quantities, such as energy and velocity, needed to obtain the thermal conductivity.

Using a Green–Kubo relation, the lattice thermal conductivity κ is computed by integrating the equilibrium time autocorrelation functions of the heat flux ***J***,
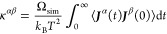
2where Ω_sim_ is the system’s volume, *k*_B_ is the Boltzmann constant, *T* is the temperature, and ⟨***J***^α^(*t*)***J***^β^(0)⟩ denotes the time autocorrelation function of ***J***. The flux consists of a kinetic and a potential part:
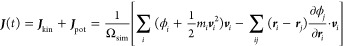
3where *m*_*i*_, ***r***_*i*_, ***v***_*i*_, and ϕ_*i*_ correspond to the mass, position, velocity, and contribution to the potential energy of atom *i*, respectively. To address typical challenges of Green–Kubo MD, such as noise, limited simulation time, and finite-size effects, we employ the approach proposed by Baroni and co-workers for time series analysis.^14,15^ In this method, the thermal conductivity κ is calculated from the zero-frequency value of the power spectrum *S*(*f*) of the heat flux, to which it is proportional. The logarithm of that zero-frequency component is then estimated as
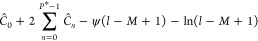


In this expression, the *Ĉ*_*n*_ (with *n* = 0, 1···) are the cepstral coefficients of ***J***, that is, the coefficients of the inverse Fourier transform of the logarithm of its spectrum, P* is a cutoff determined based on the Akaike information criterion,^43^*M* is the number of conserved fluxes in the calculation, *l* = 2 is the dimensionality of ***J***, and ψ is the digamma function. The method also provides a built-in metric of the relative uncertainty in the predicted κ. Further details can be found in (14).

We estimate the vibrational density of states (VDOS) and the projected vibrational densities of states from the power spectrum of the time autocorrelation function of the atomic velocities. More specifically, we use the implementation in the Python package pwtools,^44^ which applies a Welch window to the velocities as part of spectral density estimation.^45^

### Boltzmann Transport Equation Calculations

In order to have a reference against which to compare the MLFF results, we also obtain the thermal conductivity of the pristine bilayer from ab initio data in the framework of the Boltzmann transport equation (BTE). The details of the procedure are explained in (46). To obtain the second- and third-order interatomic force constants, the essential ingredients in these calculations, we employ Phonopy^47^ and Phono3py,^48^ respectively. We generate displaced supercell configurations based on 5 × 5 × 1 supercells for the second- and third-order calculations, with the displaced atoms offset from their equilibrium positions by 0.03 Å. The cutoff distance for interactions is set to 6 Å when calculating third-order force constants. The forces on atoms in those configurations are obtained from DFT runs using the same parameters described when discussing the generation of the data set, and the BTE is solved using Phono3py. The raw second-order force constants are postprocessed using hiPhive^49^ in order to enforce the rotational symmetry of free space, which is critical to obtain the correct quadratic behavior of the ZA branch close to the Γ point in 2D materials.^50^ In what follows, the direct ab initio results are labeled as “DFT”.

**Figure 4:**
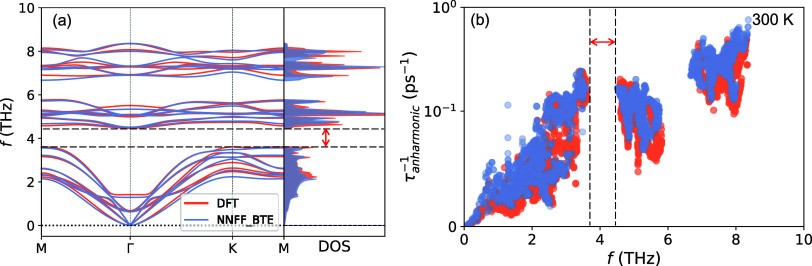
Comparison between DFT and NNFF results for two key ingredients in the BTE treatment of bilayer PtSTe: (a) harmonic phonon dispersion and density of states and (b) anharmonic scattering rates at 300 K.

Figure 4 contains a comparison between the results of DFT and NNFF when used as backends for the BTE calculation. In particular, it shows the phonon spectra, the vibrational densities of states, and the anharmonic phonon scattering rates. The phonon DOS is calculated with Phonopy using the linear tetrahedron method.^51^ Although minor differences exist, all the panels show good consistency between both methods, indicating that the trained NNFF model can predict the thermal properties of bilayer PtSTe as accurately as DFT calculations. The phonon modes of pristine bilayer PtSTe exhibit an acoustic-optical gap, indicated by the gray dashed line and the red arrows.

## Results and Discussion

**Figure 5:**
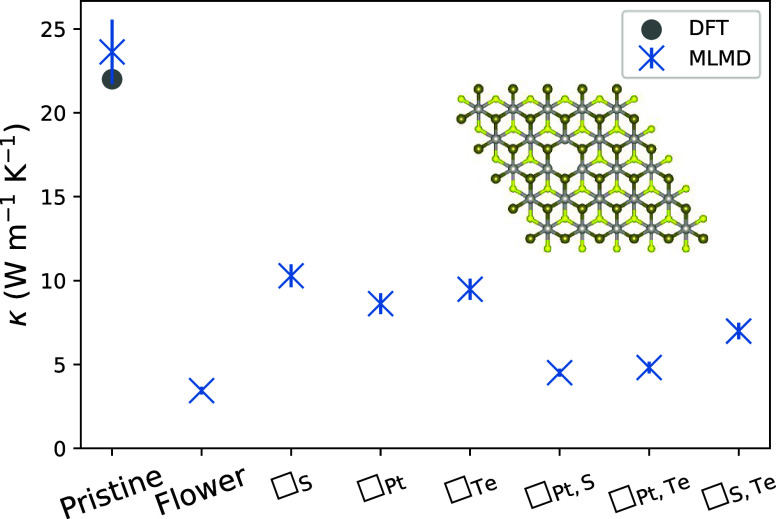
Room-temperature κ of pristine and defect-laden bilayer PtSTe using a constant defect concentration of 4.9 × 10^13^ cm^–2^.

Figure 5 shows the computed room-temperature thermal conductivity κ of bilayer PtSTe with single vacancies, double vacancies, and flower defects at a constant concentration of 4.9 × 10^13^ cm^–2^. This corresponds to a single defect in a 4 × 4 × 1 supercell, as shown in the inset. The details of the MLMD running process are shown in Figure S2 in the Supporting Information, where all κ values are shown to converge. As expected, all kinds of defects lead to a significant decrease in κ compared to the pristine structures, but there are marked differences among them. For the single vacancies, the thermal conductivities follow the order κ_□_S__ > κ_□_Te__ > κ_□_Pt__. The double vacancies depress the thermal conductivity more intensely; among those, an instance of □_Pt,S_ or □_Pt,Te_ in the supercell causes significantly lower thermal conductivities than □_S,Te_. The general trend is, thus, that heavier missing atoms are more effective at reducing κ,^52^ similar to what was recently found in Janus WSSe and MoSSe.^53^ This can be attributed to the higher low-frequency densities of states at those atoms. Finally, the flower defect yields the lowest thermal conductivity among this set.

For comparison, we also calculate the thermal conductivity of the PtSTe monolayer with the same defects in the same concentrations and at the same temperatures. The results are included in Figure S3 of the Supporting Information. As had been previously reported,^18^ the pristine monolayer has a higher thermal conductivity and, in fact, each defect-laden monolayer shows a higher thermal conductivity than the corresponding defect-laden bilayer. However, defects cause comparable percentual reductions in thermal conductivity in both systems. This shows that those defects affect both interacting layers of the bilayer, which cannot thus be regarded as independent conduction channels. Moreover, the relative efficiencies of each kind of defect for depressing the thermal conductivity follow a very similar order, the same order in the monolayer and the bilayer; in particular, a flower defect is the most powerful scatterer in both cases.

**Figure 6:**
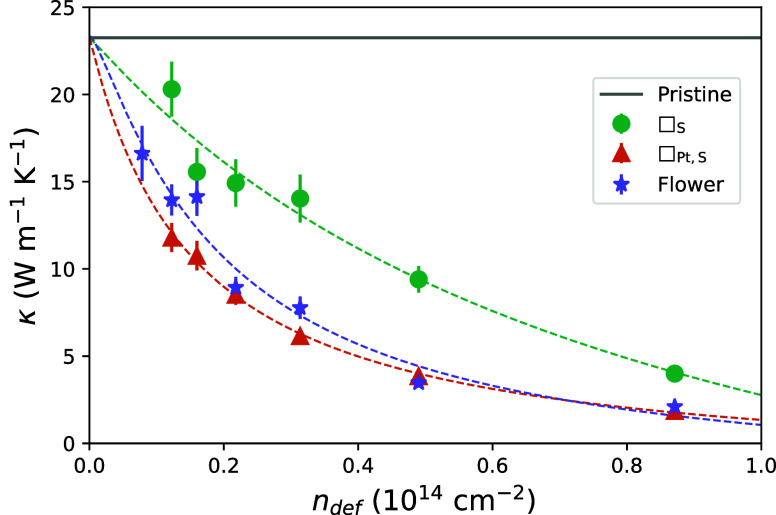
κ vs defect concentration for different defect-laden configurations of bilayer PtSTe. The dashed curves are fits to eq 4. The fitted parameters are shown in Table 2.

Figure 6 shows MLMD-calculated κ as a function of the defect concentration *n*_def_ (number of defects per unit of surface area) in bilayer PtSTe with flower defects as well as representative examples of single and double vacancies. Here, the different concentrations are achieved by changing the size of the supercell. Several factors manifest in the different trends observed for the single vacancy and the other two defect types, most importantly the balance between intrinsic three-phonon scattering and elastic phonon-defect scattering, and possible interactions between defects at higher densities.

As a first qualitative approach to this concentration dependence, we devise an interpolation between the low- and high-concentration limits of κ(*n*_def_). When *n*_def_ → 0, the thermal conductivity must tend to the pristine κ(0). On the other hand, for high concentrations, we adopt the simplistic approximation that the mean free path of each phonon, significantly lower than its intrinsic value, is limited by the characteristic distance between defects, and therefore proportional to *n*_def_^–1/2^ (although this trend would not continue indefinitely beyond the Ioffe–Regel threshold). Between those two limits, we use a simple Padé interpolation in the variable *n*_def_^1/2^:
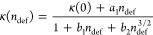
4

The fitted curves are shown in Figure 6 as a guide to the eye, and the best-fit coefficients are listed in Table 2. In the high end of the concentration range, the ratio *a*_1_/*b*_2_ gives the coefficient of the dominating *n*_def_^–1/2^ term; its value for the single vacancy is the highest and that of the flower defect is the lowest. This observation is consistent with the expected order of the scattering cross section: the flower defect scatters phonons more intensely than the double vacancy, which, in turn, is a more efficient scatterer than the single vacancy. The points follow a less regular trend at low concentrations, which can be attributed to the larger uncertainty in the results, as represented by the error bars.

**Table 2:** Parameters in Eq 4

	*a*_1_ (Wcm^2^m^–1^K^–1^)	*b*_1_ (cm^2^)	*b*_2_ (cm^3^)
□_S_	–17.20	1.27	–0.08
□_Pt,S_	–13.03	6.82	–0.21
flower	–16.85	6.36	–1.18

**Figure 7:**
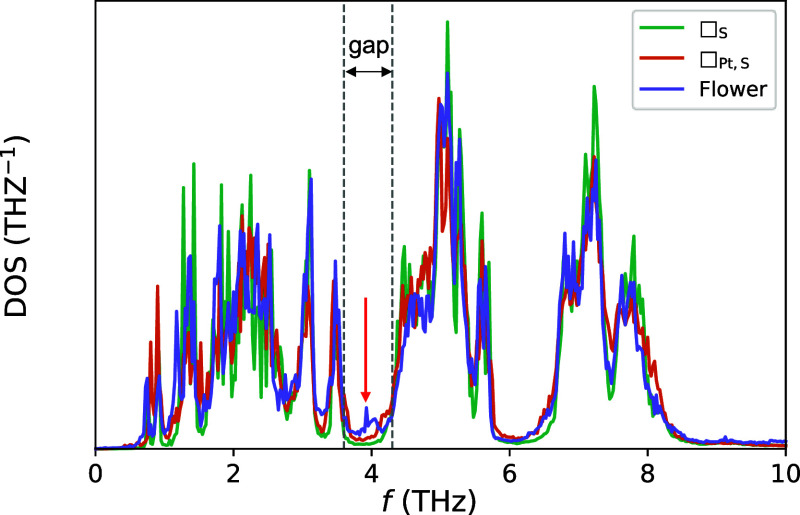
VDOS of defect-laden structures at 300 K. Dashed lines represent the phonon gap of the pristine structure, whose DOS is presented in Figure 4b. The defect concentration is set to 2.87 × 10^13^ cm^–2^.

The difference in the decreasing trends of κ with increasing *n*_def_ for different impurities is coherent with the VDOS, as shown in Figure 7. Compared with the PtSTe bilayer containing single vacancies, the ones with double vacancies and flower defects are more strongly smoothed, with lower peaks. The sequence is even more evident in the phonon gap regions; in particular, flower defects introduce a new peak in the first phonon gap, indicated by the red arrow around 4 THz in Figure 7, suggesting a significant phonon localization effect.^54^

**Figure 8:**
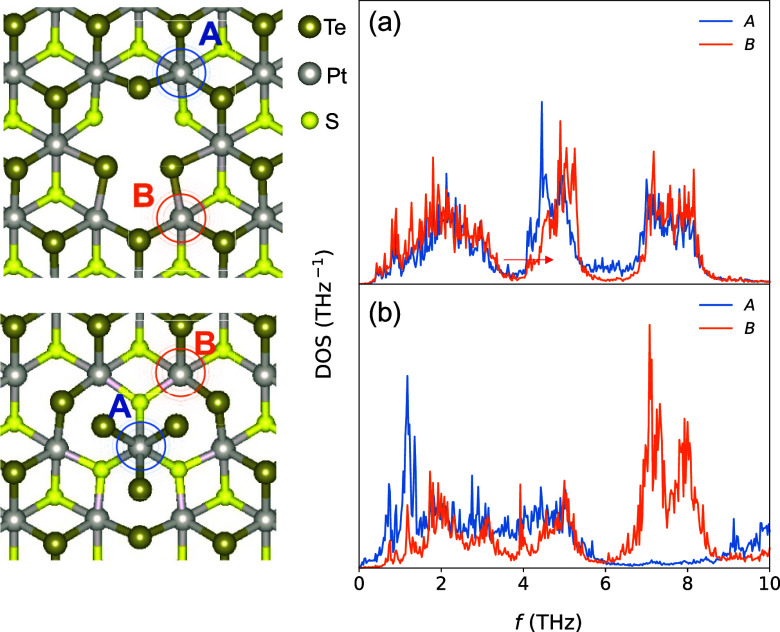
VDOS of Pt-centered atomic environments around a double-vacancy (a) and a flower (b) defect at 300 K. Each environment contains a single Pt atom and the chalcogen atoms bonded to it.

To take a closer look at the situation, in panel (a) of Figure 8 we plot a comparison between the VDOS curves of two different groups of atoms—each containing a platinum atom and the chalcogen atoms around it—near the double-vacancy defect □_Pt,S_. The platinum atom at the center of the environment labeled as A retains its full coordination, albeit with some distortion, whereas that coordination is reduced in environment B due to the missing atoms. That undercoordination leads to stiffer Pt–Te bonds and to a blue shift of the central part of the spectrum, which explains some of the significant broadening of the VDOS of the whole structure around that frequency range. A similar comparison for the flower defect is illustrated in panel (b) of the same figure. There, A and B represent the fully rotated center of the flower and a Pt-centered environment on the edge of the flower, respectively. In this case, the contrast is much clearer. While the low-frequency part of the projected VDOS of both structures is very different from that of the bulk system (and both introduce numerous states in the gap), atoms in environment A barely take part in any vibrations with frequencies compatible with the highest-lying optical branches of bulk PtSTe between 6 and 9 THz.

**Figure 9:**
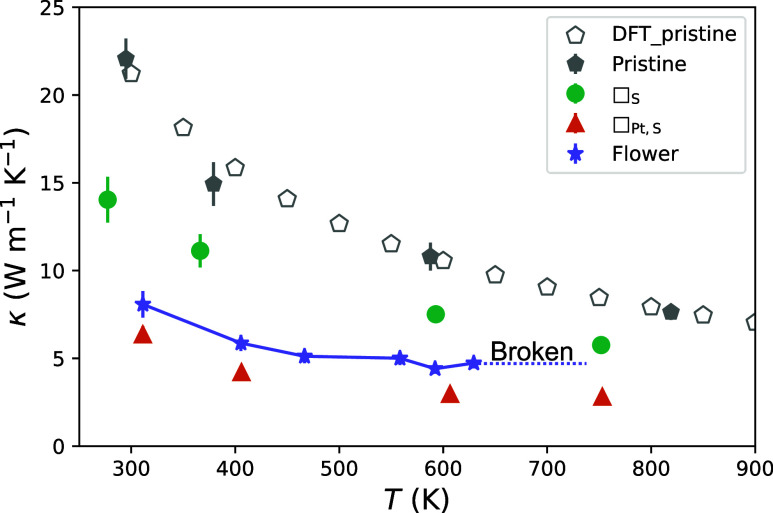
Temperature dependence of κ in bilayer PtSTe with different defects at a concentration *n*_def_ = 2.87 × 10^13^ cm^–2^.

The amount of distortion introduced by the flower defect despite not removing any atom from the structure is undoubtedly connected to its efficacy as a phonon scatterer. However, it also invites questions about its structural stability at high temperatures. Figure 9 shows the results of a series of MD simulations of defect-laden bilayer PtSTe at different temperatures with a constant defect concentration. For reference, the κ of the pristine structure is also provided: it can be seen that the latter follows a *T*^–1^ trend at high temperatures, as usual when three-phonon scattering dominates. Defects introduce temperature-independent elastic scattering^55^ and weaken the temperature dependence. This is most evident for the double vacancy and the flower defect, for which κ quickly reaches a plateau.

When *T* increases beyond 650 K, the thermal conductivity of the flower defect (κ_flower_), as predicted by MLMD, changes its trend and reaches unreasonably high values close to 10 W m^–1^ K^–1^ at 800 K. This is illustrated in Figure S4 of the Supporting Information and is attributable to the breakdown of the Pt–Te bonds in the area denoted as *A* in Figure 8. To try and rule out the possibility that this is an artifact of an NNFF trained on small displacements, we retrained it with an enriched data set. Additional configurations were sampled from the disintegrating MD trajectory, including 200 at 400 K and 200 at 800 K. The bond breaking phenomenon is still observed around *T* = 700 K, suggesting an actual instability.

## Conclusions

We have combined MD simulations with an MLFF to examine the influence of vacancies and flower defects on the thermal transport properties of bilayer PtSTe. Our findings reveal that the structure with flower defects exhibits an unexpectedly low thermal conductivity, comparable to that with double vacancies at a given defect concentration. To explore this phenomenon, we have conducted computations on the VDOS of the system and particular subsets of atoms near the defects. The results indicate a notable phonon localization effect induced by lattice distortion around the flower defect, in addition to broadening and blue shift effects. Furthermore, we have investigated the temperature dependence of the thermal conductivity of the defect-laden bilayer PtSTe. Notably, the structure with flower defects exhibits an unusual temperature dependence at high temperature, attributed to structural instability.

## Data Availability

The data sets and the coefficients of the trained models are included as part of the Supporting Information. The code of the committee-based NNFF used in this study is available at Zenodo.^56^

## Supporting Information Available

Parity plots of the NNFF over the test set, plot with a comparison between mono- and bilayers, and details of the MD simulation process for calculating the thermal conductivity in pristine and defect-laden bilayer PtSTe (PDF)
Dataset and model parameters for the trained committee of neural-network force fields (ZIP)
