# Supplementary material for: Phonon Transport in Defect-Laden Bilayer Janus PtSTe Studied Using Neural-Network Force Fields
Source: J Phys Chem C Nanomater Interfaces. 2024 Jun 22;128(26):11024–32. doi: 10.1021/acs.jpcc.4c02454 (PMC11229070; doi:10.1021/acs.jpcc.4c02454)
Supplement: Supplementary file 1 — jp4c02454_si_001.pdf [file jp4c02454_si_001.pdf]

Supporting Information for Phonon Transport in Defect-Laden Bilayer Janus PtSTe Studied Using Neural-Network Force Fields

Lijun Pan,[†,‡] Jesús Carrete,[*,¶,‡] Zhao Wang,[*,†] and Georg K. H. Madsen[‡]

†*Department of Physics, Guangxi University, Nanning 530004, China*

‡*Institute of Materials Chemistry, TU Wien, 1060 Vienna, Austria*

¶*Instituto de Nanociencia y Materiales de Aragón (INMA), CSIC-Universidad de Zaragoza, E-50009 Zaragoza, Spain*

E-mail: jcarrete@unizar.es; zw@gxu.edu.cn

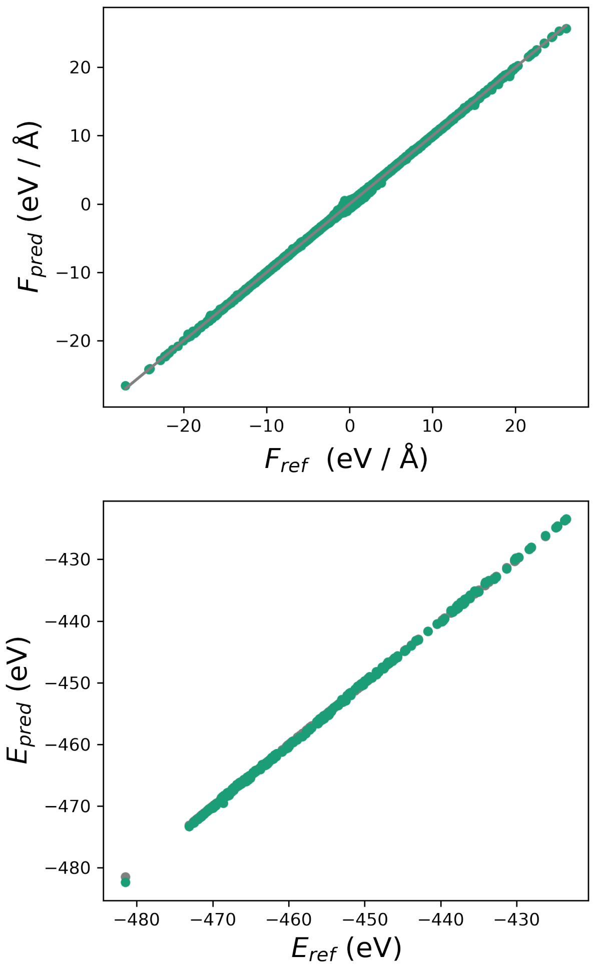

Figure S1: NNFF-predicted potential energy and atomic forces vs. reference *ab initio* data over the test subset.

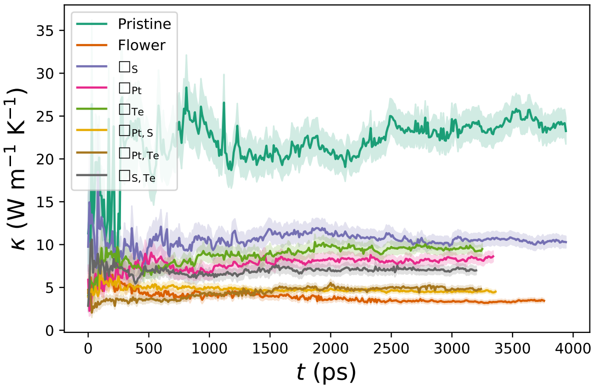

Figure S2: Time evolution of the MD-calculated lattice thermal conductivity in pristine and defect-laden bilayer PtSTe.

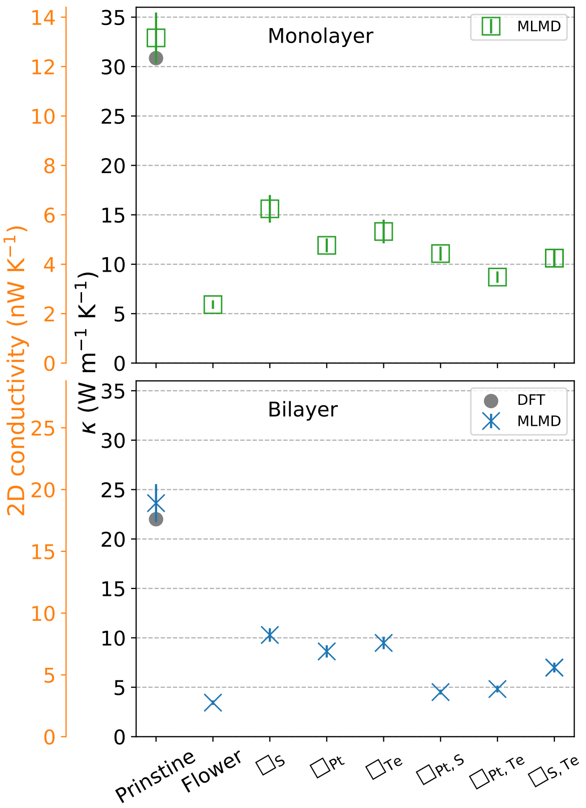

Figure S3: Thermal conductivity of pristine and defect-laden bilayer and monolayer PtSTe at room temperature with a defect concentration of 4.9×10^{13} cm^{-2}. For quasi-2D structures embedded in 3D space, there are two conceivable ways to define a thermal conductivity. In the main text we employ the same definition as for 3D crystal, as the tensor connecting the heat current per unit of cross-sectional area to the temperature gradient. However, it can also be defined as the tensor connecting the heat current per unit of cross-sectional length to the temperature gradient. The latter definition does not require assuming any conventional thickness for the structure. In this plot we present both possibilities, since the outcome of the comparison of absolute values of κ depends on the convention chosen, as the monolayer is half as thick as the bilayer.

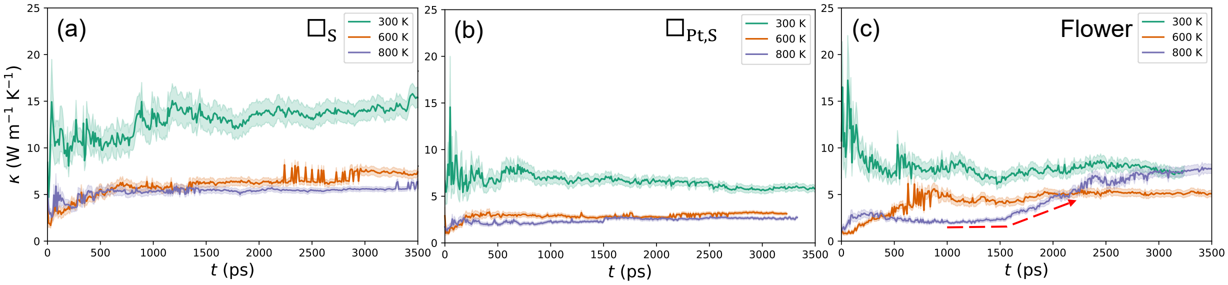

Figure S4: Time evolution of the MD-calculated lattice thermal conductivity over 3000 ps at 300, 600, and 800 K for defect-laden bilayer PtSTe with different types of defects: (a) $\square_{\mathrm{S}}$, (b) $\square_{\mathrm{Pt,S}}$, and (c) flower defect.
